# Detection of Signal Regulatory Protein α in *Saimiri sciureus* (Squirrel Monkey) by Anti-Human Monoclonal Antibody

**DOI:** 10.3389/fimmu.2017.01814

**Published:** 2017-12-14

**Authors:** Hugo Amorim dos Santos de Souza, Edmar Henrique Costa-Correa, Cesare Bianco-Junior, Márcia Cristina Ribeiro Andrade, Josué da Costa Lima-Junior, Lilian Rose Pratt-Riccio, Cláudio Tadeu Daniel-Ribeiro, Paulo Renato Rivas Totino

**Affiliations:** ^1^Laboratory for Malaria Research, Instituto Oswaldo Cruz (IOC), Fundação Oswaldo Cruz (Fiocruz), Rio de Janeiro, Brazil; ^2^Service of Primatology, Instituto de Ciência e Tecnologia em Biomodelos, Fiocruz, Rio de Janeiro, Brazil; ^3^Laboratory of Immunoparasitology, Instituto Oswaldo Cruz (IOC), Fiocruz, Rio de Janeiro, Brazil

**Keywords:** non-human primates, *Saimiri sciureus*, immune response, signal regulatory protein α, flow cytometry

## Abstract

Non-human primates (NHP) are suitable models for studying different aspects of the human system, including pathogenesis and protective immunity to many diseases. However, the lack of specific immunological reagents for neo-tropical monkeys, such as *Saimiri sciureus*, is still a major factor limiting studies in these models. An alternative strategy to circumvent this obstacle has been the selection of immunological reagents directed to humans, which present cross-reactivity with NHP molecules. In this context and considering the key role of inhibitory immunoreceptors—such as the signal regulatory protein α (SIRPα)—in the regulation of immune responses, in the present study, we attempted to evaluate the ability of anti-human SIRPα monoclonal antibodies to recognize SIRPα in antigen-presenting *S. sciureus* peripheral blood mononuclear cells (PBMC). As shown by flow cytometry analysis, the profile of anti-SIRPα staining as well as the levels of SIRPα-positive cells in PBMC from *S. sciureus* were similar to those observed in human PBMC. Furthermore, using anti-SIRPα monoclonal antibody, it was possible to detect a decrease of the SIRPα levels on surface of *S. sciureus* cells after *in vitro* stimulation with lipopolysaccharides. Finally, using computed-based analysis, we observed a high degree of conservation of SIRPα across six species of primates and the presence of shared epitopes in the extracellular domain between humans and *Saimiri* genus that could be targeted by antibodies. In conclusion, we have identified a commercially available anti-human monoclonal antibody that is able to detect SIRPα of *S. sciureus* monkeys and that, therefore, can facilitate the study of the immunomodulatory role of SIRPα when *S. sciureus* is used as a model.

## Introduction

*Saimiri sciureus*, also known as squirrel monkey, is a small species of non-human primate natively found in the tropical rainforests of South America (1, 2). As many other non-human primates (NHP), *S. sciureus* is used in diverse areas of biomedical research and, although its full genome has not yet been sequenced, the well-known close phylogenetic relationship of NHP to humans renders this model an accurate system to study biological, immunological, and pharmacologycal phenomena of medical importance (2). Indeed, *S. sciureus* has been shown to be susceptible to several human pathogens and, in this way, has been proposed as model for study the pathogenesis of malaria (3), measles (4), HTLV-associated diseases (5), BK virus infection (6), and vaginal trichomoniasis (7). Moreover, *S. sciureus* has been studied in the context of Parkinson’s disease therapy (8) and, as recommended by the World Health Organization (9), malaria vaccine candidates have been frequently tested in preclinical trials using *S. sciureus* in the last three decades (10, 11). However, the lack of specific immunological tools to assess immune response of *S. sciureus* represents a major factor limiting vaccinology and immunopathology studies using this model.

An alternative strategy to circumvent this limitation is the identification of immunological reagents directed to molecules of human immune system that also present reactivity with *S. sciureus*. In fact, a variety of anti-human monoclonal antibodies commercially available are able to satisfactorily detect surface molecules of immune cells as well as cytokines of *S. sciureus* (12–14) and other non-human primate models, such as common marmoset (*Callithrix jacchus*), rhesus macaque (*Macaca mulatta*), and chimpanzee (*Pan troglodytes*) (15–17). To the best of our knowledge, however, there is no evaluation concerning the signal regulatory protein α (SIRPα) in NHP.

Signal regulatory protein α is a transmembrane protein present in leukocytes of the myeloid lineage, including monocytes and dendritic cells (DC), which is implicated in inhibitory signaling of innate immune functions, such as phagocytosis, proinflammatory cytokine production, and DC maturation (18–20), as well as induction of programmed cell death (21). Comprehensively, SIRPα is believed to play a relevant role in the regulation of immune responses, impacting the pathogenesis of etiologically distinct diseases as well as vaccination (22–24). Nevertheless, SIRPα has not been investigated in non-human primate models. Thus, attempting to support further studies related to involvement of SIRPα in immune responses, in the present work, we evaluated by flow cytometry if monoclonal antibody directed to human SIRPα cross-reacts with peripheral blood mononuclear cells (PBMC) from *S. sciureus*.

## Materials and Methods

### Animals and Blood Samples

Seven clinically healthy *S. sciureus* monkeys from the breeding colony at the Department of Primatology of the Instituto de Ciência e Tecnologia em Biomodelos/Fiocruz, Rio de Janeiro, Brazil, were studied. Animals were male adults, aged 3–10 years, housed in accordance with the guidelines of the institutional ethical committee for animal use. For blood sample collection, animals were anesthetized with a combination of 0.1 mL midazolan and 0.4 mL ketamine and, then, 4 mL heparinized venous blood were drawn *via* femoral venipuncture. All animal experimentation was performed in compliance with the protocol reviewed and approved by the Fiocruz ethical committee (LW-9/14). Peripheral blood samples (4 mL) from five healthy human donors were also obtained by venipuncture in heparinized tubes, as approved by the Fiocruz Research Ethic Committee (46084015.1.0000.5248).

### PBMC Isolation and Antigenic Stimulation

Peripheral blood mononuclear cells were isolated from *S. sciureus* whole blood through density gradient centrifugation using Histopaque-1077 (Sigma). Cells were washed twice in RPMI-1640 medium (Sigma) containing 2.05 mM l-glutamine, 25 mM Hepes, and 2.0 g/L sodium bicarbonate and, then, resuspended in RPMI medium supplemented with 200 U/mL penicillin (Gibco), 200 mg/mL streptomycin (Gibco), and 10% inactivated fetal calf serum (Gibco). Cells (2.5 × 10^5^) were assayed *ex vivo* or after 24 h stimulation with *Escherichia coli* lipopolysaccharides (LPS, 5 µg/mL, Sigma) in 96-well culture plates (Falcon) at 37°C in 5% CO_2_.

### Flow Cytometry Assay

Detection of SIRPα in *S. sciureus* PBMC was assayed by flow cytometry using allophycocyanin (APC)-conjugated anti-human SIRPα monoclonal antibody purchased from eBioscience (isotype: mouse/IgG2a, clone: 15-414). Cells (2.5 × 10^5^) were washed in phosphate saline buffer (PBS) and, subsequently, incubated at 4°C for 30 min in PBS containing 10% fetal bovine serum (FBS) to reduce non-specific staining. After incubation, cells were stained with 2.0 µL anti-SIRPα monoclonal antibody or APC-conjugated isotype control (eBioscience) at 4°C for 40 min in 100 µL PBS containing 1% FBS. Cells were washed twice and, finally, analyzed by a FACSVerse flow cytometer (Becton Dickinson). In parallel, anti-SIRPα monoclonal antibody was tested *ex vivo* with PBMC obtained from blood human samples, as described in Section “PBMC Isolation and Antigenic Stimulation.”

### Computer-Assisted Analysis of Sequence Alignment and Potential B-Cell Epitopes

To detect SIRPα protein homology among several primate species, protein BLAST were done and protein sequences of *Homo sapiens* (AAH26692.1), *P. troglodytes* (JAA44167.1), *C. jacchus* (JAB51896.1), *Macaca fascicularis* (XP_015313155.1), *Gorilla gorilla* (XP_004061735.2), and *Saimiri boliviensis* (XP_010350139.1) were analyzed. Multiple alignment CLUSTAL OMEGA, distance matrix, and the phylogenetic tree were done using the Megalign Pro 15 (Lasergene DNASTAR) program and the circular map of protein alignment was generated using the software GenVision 15 (Lasergene DNASTAR). The prediction of linear B-cell epitopes was carried out using the web server BepiPred. For each input FASTA sequence of extracellular domain of SIRPα, the server outputs a epitope prediction score for each amino acid. The recommended cutoff of 0.35 was used to determine potential B-cell linear epitopes, ensuring sensibility of 49% and specificity of 75%. Linear B-cell epitopes of SIRPα extracellular domain of *H. sapiens* and *S. boliviensis* were predicted to be located at the residues with the highest scores in at least nine consecutive amino acids.

## Results and Discussion

Signal regulatory protein α has been studied by flow cytometric analysis in both human (25, 26) and animal models, i.e., mice, rats, and cattle (27–29), but the frequency and distribution of SIRPα-positive cells in peripheral blood has not been reported. Thus, to investigate the reactivity of anti-human SIRPα monoclonal antibody with *S. sciureus* PBMC by flow cytometry; we first evaluated anti-SIRPα staining profile in PBMC obtained from five normal healthy human donors.

**Figure 1:**
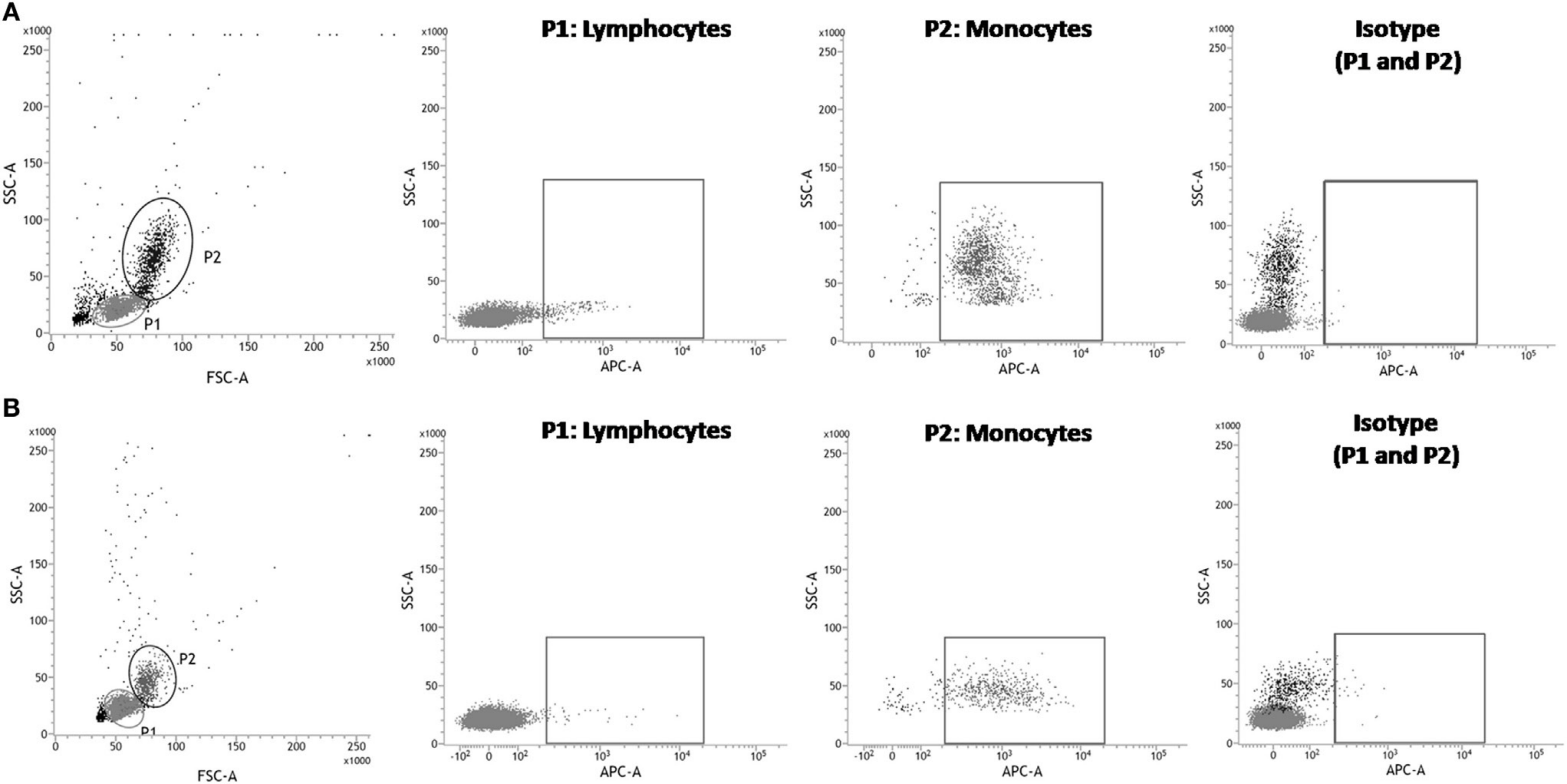
Flow cytometry analysis of anti-human signal regulatory protein α (SIRPα) monoclonal antibody cross-reactivity with *Saimiri sciureus* cells. Peripheral blood mononuclear cells (PBMC) were isolated from human or *S. sciureus* whole blood, stained with anti-SIRPα monoclonal antibody or isotype control and, then, analyzed by flow cytometry. Reactivity of anti-SIRPα antibodies [allophycocyanin (APC)] with human **(A)** and *S. sciureus* **(B)** PBMC was evaluated gating lymphocytes (P1) or monocytes populations, as defined by forward scatter (FSC) and sideward scatter (SSC) parameters.

**Figure 2:**
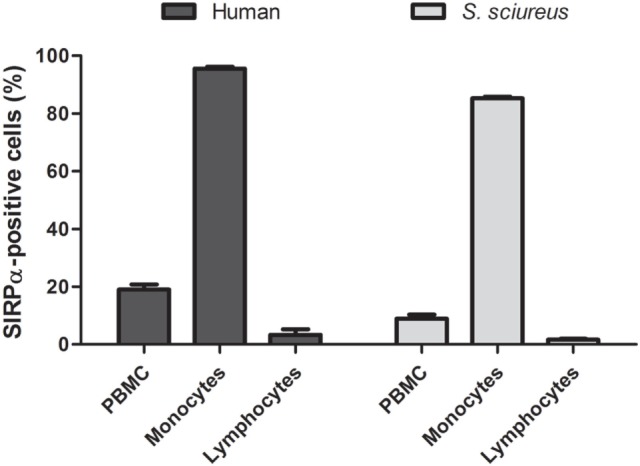
Frequency of signal regulatory protein α (SIRPα)-positive cells in peripheral blood mononuclear cells (PBMC) samples from humans and *Saimiri sciureus* monkeys. PBMC were isolated from whole blood, stained with anti-human SIRPα monoclonal antibody and, then, analyzed by flow cytometry. Cells presenting SIRPα were quantified considering three main cells populations by morphological criteria: total PBMC, monocytes, and lymphocytes, as shown in Figure 1. Data represent mean ± SEM for five humans and seven monkeys.

Signal regulatory protein α is known as an immune inhibitory receptor present in leukocytes of the myeloid lineage and, therefore, it is expected that SIRPα in PBMC population is mainly detected on surface of cells showing monocyte morphology by size and granularity analysis in flow cytometry using forward scatter and sideward scatter parameters (30). Indeed, an elevated percentage (95.55 ± 1.16%) of SIRPα-positive cells was observed in the human monocyte population, while only 3.27 ± 3.38% cells presented SIRPα in the lymphocyte population (Figures 1A and 2). Moreover, SIRPα-positive cells corresponded to 18.98 ± 3.12% of total PBMC, agreeing with the frequency of total myeloid innate immune cells found in human PBMC samples, which manly comprises monocytes and DC (31, 32).

Subsequently, anti-human SIRPα monoclonal antibody was tested against *S. sciureus* cells. Previous reports demonstrated that different immune cell surface receptors as well as cytokines of *S. sciureus* can be detected by a range of antibodies directed to human (12–14) and, in the same way, we observed that anti-human SIRPα antibody cross-reacted with cell surface of *S. sciureus* PBMC. As shown in Figure 1B, the profile of anti-SIRPα staining in PBMC from *S. sciureus* was similar to that observed in human samples. SIRPα-positive *S. sciureus* cells corresponded to 8.92 ± 3.65% of total PBMC and 1.59 ± 1.03% of the lymphocyte population, while an increased frequency of SIRPα-presenting cells (85.27 ± 1.41%) was observed in monocytes population (Figure 2). These data suggest that anti-human SIRPα antibody recognizes a specific antigen present on surface of *S. sciureus* innate immune cells, possibly the cognate of human SIRPα in *S. sciureus*.

Although the cross-reactivity of antibodies cannot indicate *per se* the degree of homology between proteins across phyla, an increased similarity (>90%) has been shown through molecular approaches between human, *S. sciureus*, and other NHP concerning nucleotide sequence of genes coding for many cytokines (33, 34) as well as dopamine transport (35) and, therefore, it was already possible to quantify gene expression of 12 *S. sciureus* cytokines (IL-1A, IL-2, IL-4, IL-5, IL-6, IL-10, IL-12B, IL-17, IFN-β, IFN-γ, LTA, and TNF) by commercially available real-time PCR assays using predesigned human gene-specific primers and probes (14). Moreover, genomic studies demonstrate the presence of SIRPα gene in a vast group of animals, from cats to NHP, supporting that SIRPα is a ubiquitous molecule of innate immune system of mammalians (36, 37). In the case of *S. sciureus* SIRPα, however, there are no molecular data available, i.e., neither genome nor SIRPα gene was reported yet, limiting the analysis of *S. sciureus* SIRPα homology with their cognates in other primates.

**Figure 3:**
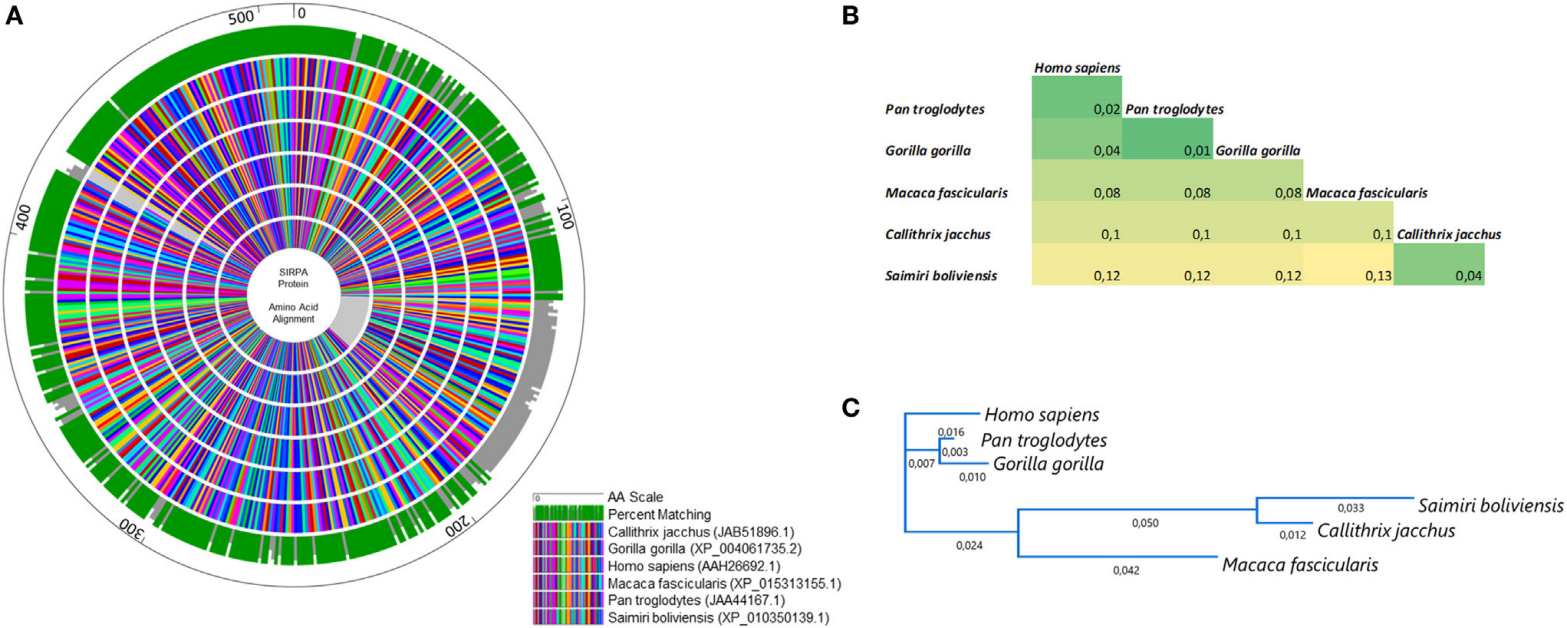
Homology analysis of signal regulatory protein α (SIRPα) across primates. **(A)** Circular alignment of amino acid sequences of SIRPα protein in human and five non-human primates (*Pan troglodytes, Gorilla gorilla, Macaca fascicularis, Callithrix jacchus, Saimiri boliviensis*). The outer circle shows the amino acid scale. Green and gray bars on the second circle show the percent matching among all sequences used in the analysis. Inner circles show the sequence alignment in which each amino acid was represented by a different color. **(B)** Pairwise distance among all primates studied and **(C)** phylogenetic tree based on SIRPα protein alignments.

**Figure 4:**
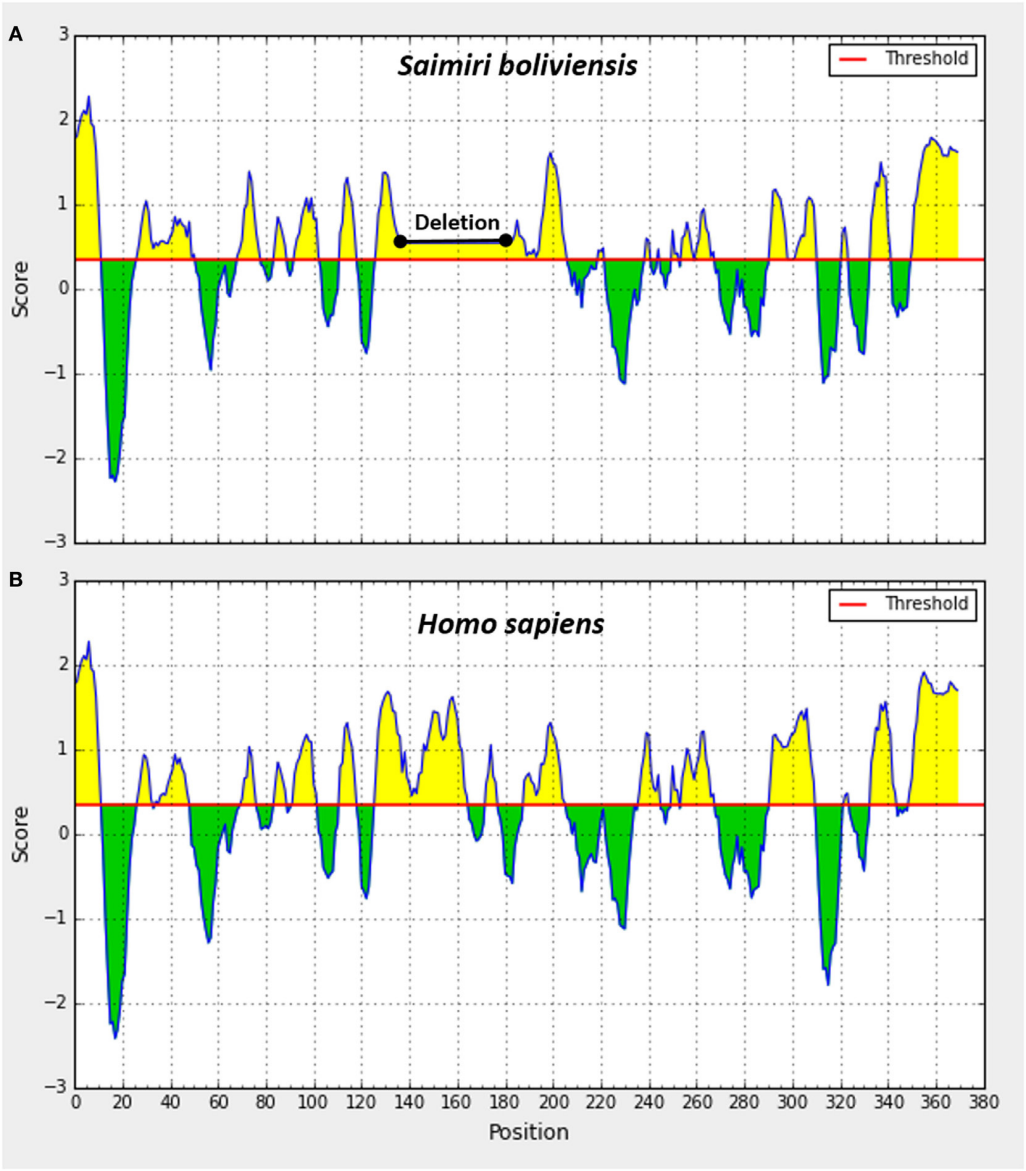
Prediction of linear B-cell epitopes in extracellular domain of signal regulatory protein α protein in *Saimiri* **(A)** and *Homo sapiens* **(B)**. Linear B-cell epitopes were predicted to be located at the residues with the scores above 0.35 (yellow) and regions not predicted to be B-cell epitopes are under the threshold (green). The epitope score represents the average of the scores of least nine consecutive amino acids above the cut-off, and the sequences with higher mean values were detected as potential linear epitopes.

In this scenario, to confirm that the cross-reactivity herein detected was a natural consequence of the similarity of SIRPα protein across primates, we aligned the amino acid sequences of SIRPα from six different primate species including *Saimiri* and *Homo sapiens* (Figure 3). As expected, a significant degree of identity was observed across the primates, which showed a complete matching in 72% of all sequences analyzed (Figure 3A). The homology rate ranged from 87% (*M. Fascicularis* vs. *S. boliviensis*) to 99% (*P. troglodites* vs. *G. gorilla*) and human SIRPα showed a high identity with its orthologs, ranging from *88% in S. boliviensis* to 98% in *G. gorilla*, despite the deletion of 58 amino acid present in *S. boliviensis* sequence, which was determinant to reduce the homology rate (Figures 3A,B). Since the amino acid sequence to which the commercial anti-human SIRPα monoclonal binds is not available, we also checked if the deletion in *S. boliviensis*, which is taxonomically the closest to *S. sciureus* among the NHP species studied herein, could potentially influence the antibody recognition. In this way, we verified the potential epitopes shared between the *H. sapiens* and *S. boliviensis* through analysis of linear B-cell epitopes in SIRPα extracellular domain and we observed at least 10 B-cell epitopes that can be targeted by antibodies (Figure 4). Importantly, all of these regions were shared by both species, indicating that anti-human SIRPα antibodies can target SIRPα of *Saimiri* monkeys in a similar way to its orthologous in human.

**Figure 5:**
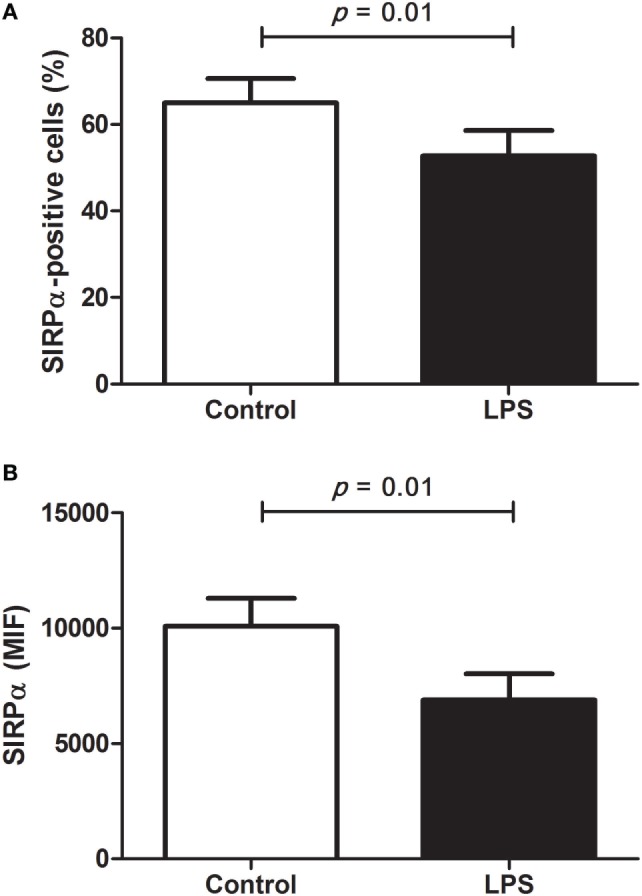
Modulation of signal regulatory protein α (SIRPα) levels by lipopolysaccharides (LPS) in *Saimiri sciureus* cells. Peripheral blood mononuclear cells (PBMC) of *S. sciureus* monkeys (*n* = 7) were incubated for 24 h in presence or absence (control) of LPS and, then, SIRPα was detected in monocytes population by flow cytometry using anti-human monoclonal antibody. **(A)** Frequency of SIRPα-positive cells in PBMC and **(B)** levels of SIRPα present on cell surface of SIRPα-positive cells (monocytes), as measured by mean fluorescence intensity. Data (mean ± SEM) are representative of two separate experiments. Statistical difference was tested by paired *t*-test in GraphPad Prism 5.0 software and *p* < 0.05 was considered significant.

Thus, to better study the capacity of anti-human antibodies to detect *S. sciureus* SIRPα, we additionally evaluated the levels of this immune receptor on surface of PBMC after stimulation with LPS. It has been described that pathogen-associated molecular patterns present modulatory effects on SIRPα levels in macrophages and DC and, in this context, LPS was recognized as a negative modulator (24, 38, 39). Indeed, analyzing monocytes population by flow cytometry, which mainly includes innate immune cells present in PBMC, we found that the anti-human SIRPα monoclonal antibody was able to identify a significant reduction not only in the frequency of SIRPα-positive cells but also in the levels of SIRPα present on the surface of these cells after LPS stimulation (Figure 5). Despite LPS-mediated regulation of SIRPα expression has not been investigated in human or NHP PBMC, decreased levels of SIRPα on the surface of peripheral blood monocytes were found in LPS-treated pigs and it was already reported a downregulation of SIRPα gene expression in cultured primary mouse microglia following LPS-stimulation (40, 41), agreeing with our data on PBMC and, consequently, supporting that anti-human SIRPα antibodies can recognize SIRPα of *S. sciureus*, whose levels were downmodulated by LPS in monocyte population.

Collectively, the flow cytometry assays showing that SIRPα-positive cells are similarly present and distributed in PBMC of human and *S. sciureus*, together with observation by computed-based analysis that SIRPα has a high degree of conservation across primates, with the presence of conserved B-cell epitopes in the extracellular domain between humans and the *Saimiri* genus, strongly indicate that anti-SIRPα antibodies directed to humans can detect SIRPα of *S. sciureus*. Take into account the role of SIRPα in the negative regulation of immune responses, we believe that further studies in *S. sciureus* or other non-human primate models, exploring SIRPα signaling with anti-human antibodies, may help the understanding of the immunopathogenesis of diseases, such as cancer, neurodegenerative disorders, and infectious diseases, and, consequently, contribute to the development of therapeutic and vaccinal strategies that mitigate their impact in public health.

## Ethics Statement

This study was carried out in accordance with the recommendations and approved by the Fiocruz Ethics Committee on Animal Use (CEUA Licence LW-9/14).

## Author Contributions

HS and EC-C performed the experiments and helped PT in drafting the manuscript. CB-J and MA carried out animal manipulation and helped in the experiments. LP-R performed the experiments and helped in the computed-based analysis. JL-J performed the computed-based analyses and reviewed the manuscript. CD-R reviewed the manuscript. PT performed data analysis and reviewed the manuscript.

## Conflict of Interest Statement

The authors declare that the research was conducted in the absence of any commercial or financial relationships that could be construed as a potential conflict of interest.
